# Management of Esophageal Perforation in Adults

**DOI:** 10.4021/gr263w

**Published:** 2010-11-20

**Authors:** Lileswar Kaman, Javid Iqbal, Byju Kundil, Rakesh Kochhar

**Affiliations:** a Department of General Surgery, Post Graduate Institute of Medical Education and Research, Chandigarh, India; b Department of GI Surgery, Lakeshore Hospital, Cochin, Kerala, India; c Department of Gastroenterology, Post Graduate Institute of Medical Education and Research, Chandigarh, India

**Keywords:** Esophagus, Esophageal perforations, Esophagectomy, Boerhaave’s syndrome

## Abstract

Perforation of esophagus in the adult is a very morbid condition with high morbidity and mortality. The ideal treatment is controversial. The main causes for esophageal perforation in adults are iatrogenic, traumatic, spontaneous and foreign bodies. The morbidity and mortality rate is directly related to the delay in diagnosis and initiation of optimum treatment. The reported mortality from treated esophageal perforation is 10% to 25%, when therapy is initiated within 24 hours of perforation, but it could rise up to 40% to 60% when the treatment is delayed beyond 48 hours. Primary closure of the perforation site and wide drainage of the mediastinum is recommended if perforation is detected in less than 24 hours. Treatment option for delayed or missed rupture of esophagus is not very clear and is controversial. Recently a substantial number of patients with esophageal perforation are being managed by nonoperative measures. Patients with small perforations and minimal extraesophageal involvement may be better managed by nonoperative treatment Major prognostic factors determining mortality are the etiology and site of the injury, the presence of underlying esophageal pathology, the delay in diagnosis and the method of treatment. For optimum outcome for management of esophageal perforations in adults a multidisciplinary approach is needed.

## Introduction

Esophageal perforation is a surgical emergency associated with high morbidity and mortality. Consensus regarding the appropriate management of this life-threatening condition is lacking. The reported mortality from treated esophageal perforation is 10% to 25%, when therapy is initiated within 24 hours of perforation and it is 40% to 60% when the treatment is delayed [1-6]. The reason for this manifold increase in mortality is due to the unique anatomical configuration and location of the esophagus, which allows bacteria and digestive enzymes easy access to the mediastinum, leading to the development of severe mediastinitis, empyema, sepsis, and multiple organ dysfunction syndromes [7]. Moreover, the rarity of this condition and its nonspecific presentations lead to diagnostic and treatment delay in more than 50% of perforations [6]. Etiological factors are iatrogenic, traumatic, spontaneous and foreign bodies [7-10]. Primary closure and wide drainage of the mediastinum is the treatment of choice if detected in less than 24 hours [1-17]. Treatment option for delayed or missed rupture is not clear and is controversial [18-37]. Major prognostic factors are the cause and site of the injury, the presence of underlying esophageal pathology, the delay in diagnosis and the method of treatment [1-14].

### Etiology

Iatrogenic perforation is the leading cause of esophageal perforations [1-8] (Table 1). Iatrogenic causes account for around 70% of esophageal perforations [1, 3, 6, 8]. Endoscopic procedures are the most common cause of iatrogenic esophageal perforation. The reported risk for diagnostic esophagogastrodudenoscopy is 0.03%. The risk of perforation increases when therapeutic procedures are performed at the time of endoscopy. The reported risk for perforation is 0.5% in esophageal dilation, 1.7% in esophageal dilation for achalasia, 1-6% for endoscopic variceal sclerotherapy, 5% for endoscopic laser therapy, 4.6% for photodynamic therapy and esophageal stent placement carries a risk of 5-25% [1-8]. In diagnostic endoscopy the cervical esophagus at the cricopharynx is the most common site for injury [1, 3, 6]. Endoscope causes perforation by either piercing or shearing near the pharyngoesophageal junction where the wall is weakest. During endoscopic biopsy and therapeutic endoscopy mid and distal esophageal perforations occur [1, 3, 6, 8]. Other causes of iatrogenic esophageal perforation include nasogastric tube insertion, difficult endotracheal intubation, minitracheostomy, surgery of the mediastinal organs including resection of lung cancer, blind dissection of the abdominal esophagus, operations on the cervical spine, thyroidectomy, and palliative intubation, stenting, or laser treatment of esophageal tumors [1-8]. Another interesting reported cause of esophageal perforation is transesophageal echo, which is associated with 0.1% to 0.3% perforation rate [38].

**Table 1:** Etiology of Esophageal Perforations

Endoscopic
- Diagnostic endoscopy
- Endoscopic biopsy
- Endoscopic dilatations
- Variceal Sclerotherapy
- Endoscopic laser therapy
- Endoscopic Photodynamic therapy
- Endoscopic Stent Placement
Nasogastric tube placement
Endotracheal intubations
Transesophageal echocardiography
Minitracheostomy
Foreign bodies-
Bones, dentures, button batteries
Trauma
- Blunt
- Penetrating
- Sword swallowing
Spontaneous or Boerhaave’s syndrome
Caustic agents
- Acid and alkali
Severe Reflux and Mallory-Weiss tear
Infective causes
- Candida
- Herpes
- Syphilis
- Tuberculosis
- Immunodeficiency status
Non esophageal surgery –
Mediastinal and cervical –Thyroid, Lung, spine and mediastinal tumors
Malignancy of esophagus, Lung and other mediastinal structures

Esophageal perforations may occur due to trauma to the chest and upper abdomen [10-12, 39]. Penetrating injury may be due to gunshot or stab wound. Blunt trauma is also a major cause of esophageal perforation [12, 39]. Motor vehicle accident causes blunt esophageal perforations and 82% of these occur in the cervical and upper thoracic esophagus, perhaps because that is just distal to where the esophagus is fixed [39].

Boerhaave’s syndrome or spontaneous perforation of esophagus is induced by straining and vomiting [13-15]. There is generally a history of resisting vomiting, but it has also been reported after weight lifting, coughing and childbirth. Ruptures usually occur in the left posterior aspect of the lower esophagus and are more frequent in males [13-15]. The delay in diagnosis and treatment in this condition is associated with poor survival [13-15, 40-42]. Foreign bodies, usually bones: chicken, fish, pigeon, rabbit and pork causes esophageal perforations [11, 8, 12, 16, 27, 43]. They puncture the esophageal wall directly or can cause perforation by pressure necrosis ultimately leading to perforation. Dentures are also important cause of esophageal perforations [8, 16, 27, 43]. Other notable foreign bodies include button batteries, which require urgent retrieval because of their alkaline contents [43, 44]. Caustic agents (both acid and alkali) cause injury and perforation of esophagus [43-48]. Alkaline material accounts for most cases of caustic ingestion in the developed world, whereas acid ingestion appears to be more common in developing countries, like India, where hydrochloric acid and sulfuric acid are easily accessible [47, 48]. Esophageal perforation due to blister wrapped tablets has been reported [49]. Other conditions like severe reflux and candida, herpetic and immunodeficiency infections and malignancy can also cause perforations. Mallory-Weiss tear can perforate esophagus due to rapid increases in intragastric pressure against a closed pylorus [12]

### Presenting features

Presenting features depend on the site of the perforations, the etiological factors and time of presentations [1, 2, 6, 8, 12]. Pain is present in about 80% of patients, usually referring directly to the site of perforation [1, 2, 16]. Other symptoms are vomiting, hematemesis, dysphagia, tachypnea, cough and fever [1, 2, 8, 12]. The typical presentation of spontaneous esophageal rupture is severe vomiting or retching followed by acute, severe chest or epigastric pain [13-15]. The presence of pain in the neck, upper back, chest, or abdomen, dysphagia, odynophagia, dysphonia or dyspnea and fever following esophageal instrumentation should raise suspicion for perforation of the esophagus [1-3, 6, 12, 16]. The history of foreign body or caustic agent ingestion followed by the above symptoms indicates esophageal perforation until proven otherwise. The signs for esophageal perforations are mostly nonspecific. But most of the time there is tachycardia; hypotension, shock, fever, subcutaneous emphysema, pneumothorax and hemothorax are present [1-8, 12, 16]. Subcutaneous emphysema is present in up to 60% of perforations but requires at least an hour to develop after the initial injury [8, 12, 16]. In spontaneous esophageal perforation the classical Mackler triad, consisting of vomiting, chest pain, and subcutaneous emphysema is present in about 50% of cases [13-15, 40-42]. In cervical perforation there is pain in the neck with neck stiffness due to esophageal attachment to the prevertebral fascia limiting spread of oropharyngeal soilage [1]. Surgical emphysema is also typically seen in case of cervical perforation. In case of thoracic perforation there is severe chest pain with features of mediastinitis and pneumo or hemothorax. In lower esophageal perforations there may be signs of peritonitis. Abdominal pain may radiate to back if there is collection in the lesser sac. These symptoms vary according to the etiology and the time of presentations. In cases of delayed presentation, patients may be critically ill and may present with gross sepsis and multiple organ dysfunction syndromes [8, 13-15].

## Diagnosis

The essential attribute of the diagnostic approach to esophageal rupture is the maintenance of a high index of suspicion. Any patient who presents with pain or fever following forceful vomiting, esophageal instrumentation, or chest trauma should be aggressively evaluated, with the aim of ruling out perforation of the esophagus [1-8]. The signs and symptoms of early esophageal perforations may be very subtle and can be misleading. If cervical esophageal perforation is suspected, a lateral neck X-ray may demonstrate air in the prevertebral facial planes. In thoracic or intra-abdominal esophageal perforation, posterior and lateral chest radiographs, and upright abdominal series should be obtained [50, 51]. Pneumomediastinum, subcutaneous emphysema, mediastinal widening, or a mediastinal air-fluid level may be seen in the chest x-ray [50, 52]. Pneumothorax may be present in up to 77% of the time and it occurs when there is violation of the mediastinal pleura in 70% of the time it is on the left, 20% on the right and 10% bilaterally [50, 52]. Hydropneumothorax on the left is seen in patients with distal third esophageal perforations [53]. Once there is suspicion of esophageal perforation in the chest x-ray, a contrast esophagogram should be performed immediately [1-3]. There is controversy regarding use of water-soluble contrast agent (Gastrografin) because of its moderate sensitivity (60-70%) [1-8]. Negative scan always does not exclude perforation, especially in the cervical esophagus because of the rapid transit of the thin contrast. Contrast esophagography using a water-soluble agent initially followed by a barium study if the initial result is negative. It represents the most reliable test for demonstrating the presence and location of a perforation. Dilute barium study may reveal the primary area of leakage and determines whether the perforation is confined to the mediastinum or communicates freely with the pleural or peritoneal cavities, which has got significant bearing on the subsequent management. There is a concern regarding severe inflammatory response in tissues, most notably a mediastinitis. A contrast-enhanced CT scan of the chest should be performed if there is problem in getting a contrast esophagogram or in case of negative study despite high clinical suspicion or to rule out alternative diagnosis [54, 55]. Perforation may be suggested by mediastinal air, extravasated luminal contrast, periesophageal fluid collections, pleural effusions, or actual communication of an air-filed esophagus with an adjacent mediastinal air-fluid collection [54, 55]. If a perforation is suspected during an endoscopic procedure, careful inspection of the esophagus without air insufflation is warranted before taking out the endoscope but is not recommended as a primary diagnostic procedure as insufflated air can cause further dissection of the perforation [1-8, 56]. The other diagnostic modalities that may be used are MRI to rule out dissection of aorta [52]. Ventilation perfusion (V/Q) scan and CT scanning of the lungs to rule out pulmonary embolism [52, 55]. ECG may exclude myocardial infarction or associated cardiac abnormalities.

## Management

The appropriate management of esophageal perforation is a controversial issue [1-8, 12, 16] (Table 2). Early diagnosis, in less than 24 hours is vital to good outcomes. The mortality is 10% with early diagnosis and appropriate treatment but the mortality is up to 50% with late diagnosis [1-8, 12, 16-18]. Most of the iatrogenic perforations often noted immediately during endoscopic instrumentation, results in improved outcome [3, 17, 24, 31, 36, 37]. The choice of treatment depends on the etiology, site of perforation, general physical condition of the patient and the extent of contamination as determined by radiology [1-8, 12, 14, 16, 18, 57, 58]. The treatment also depends on the status of the esophagus: perforation in a healthy esophagus and perforation with a preexisting underlying intrinsic esophageal disease causing distal obstruction need different approach [8, 28, 30]. Nonoperative treatment is appropriate when esophageal perforation is encountered late [1-5, 36, 37]. Surgery is the mainstay of treatment, but recently there has been a trend toward more non operative management [1-8, 36, 37, 59-61]^.^ Treatment should be started as early as possible and that should include intravenous fluid, nothing by mouth, broad spectrum antibiotics, narcotic analgesics, total parenteral nutrition, and decision regarding surgical closure versus non operative management [1-8, 12, 14, 16, 17]. Patients with hemodynamic instability or any degree of airway compromise should undergo treatment in an intensive care setting with complete resuscitative facilities, including emergency airway equipment and artificial respiratory support.

**Table 2:** Diagnosis of Esophageal Perforations

History
Clinical examinations
Radiology Plain
- Neck X-ray lateral view
- Chest X-ray PA view
- Abdominal X-ray erect
Radiology Contrast
- Gastrografin study(water soluble contrast)
- Thin barium swallow study
- CT scan of chest and abdomen with oral contrast
- MRI chest and abdomen
- Ventilation perfusion (V/Q) scan
ECG

## Nonoperative Management

Recent evidence indicates that a substantial number of patients with esophageal perforation can be managed by nonoperative measures [1-5, 8, 36, 37, 59-62]. Patients with small well-defined tears and minimal extraesophageal involvement may be better managed by nonoperative treatment [59, 60]. The criteria for nonoperative management was initially described by Cameron et al in 1979 and modified by Altorjay in 1997 [59, 60]. These include: early diagnosis or delayed diagnosis with contained leak, perforation not in the abdomen, contained perforation in the mediastinum, content of the perforation draining back to the esophagus, perforation does not involve neoplasm or obstruction of the esophagus, absence of sepsis, presence of experienced thoracic surgeon and contrast imaging in the hospital [59, 60]. Most of the recent iatrogenic perforations or late postemetic esophageal perforation may be managed by nonoperative management [3, 7, 24, 31, 36, 37]. Many authors believe that if treatment is delayed for more than 24 hours after the perforation, the modality of treatment really does not influence the outcome and most cases can be managed by nonoperative treatment [31, 36, 37]. Nonoperative treatment includes large bore intravenous access, supplemental oxygen and cardiopulmonary monitoring in a critical care setting. Patient should be kept nil per oral and should have a nasogastric tube placed to clear gastric contents and limit further contamination. Broad spectrum intravenous antibiotics should be instituted as early as possible and should be given for minimum of 7 – 10 days. Adequate analgesia including narcotic analgesia should be provided to control pain and discomfort, but it should be used judiciously in hypotensive patients. Intercostal chest tube should be placed to decompress the chest as and when necessary. Total parenteral nutrition should be instituted if a prolonged course is anticipated. Percutaneous gastrostomy may also be considered. Recently endoscopic placements of removable covered esophageal stents have been described in the care of patients with esophageal perforation with excellent results [63-67]. By using removable Polyflex esophageal stents both primary and secondary esophageal leaks are being treated with reduced hospital stay, fewer adjunctive procedures and early resumption of oral diet [23, 65, 66]. Stent migration is a problem and must be recognized because it may cause gastric outlet obstruction after lodgment at the pylorus. Fibrin sealant has also been used in treatment of esophageal perforation [25]. Successful endoscopic closure of esophageal perforation with metallic clips has been reported for perforations associated with instrumentation, foreign body ingestion and Boerhaave’s syndrome. This mode of treatment is suitable only for selected patients with small (≤ 1.5 cm) clean perforation and minimal symptoms of infection. Although the length of time between the occurrence and the diagnosis of perforation is an important prognostic factor, recent reports advocated clipping of mature perforation in special circumstances [68, 69]. Repeated and regular contrast study should be utilized to ascertain the progress of the treatment. Early consultation and active involvement of experienced esophageal or thoracic surgeon is a must during the nonoperative conservative management. Shifting the patient may be required to a tertiary care facility if these facilities are not available at the presenting hospital. Any signs and symptoms of sepsis during the course of nonoperative management warrant immediate surgical intervention. Respiratory complications like pneumothorax, mediastinal emphysema, and respiratory failure are also indications for surgical intervention [59, 60].The mortality for nonoperative management of esophageal perforations is 20 to 38% [1-8, 11, 12]. But in some center with carefully selected patients the reported mortality from nonoperative management has been zero [3, 13] (Table 3).

**Table 3:** Treatment Options for Esophageal Perforations

Operative	Non operative
Primary closure	Conservative management
Primary closure with buttressing of repair with	Esophageal stenting
- Pleural flap	Fibrin glue applications
- Pericardial fat pad	Endoclip application
- Diaphragmatic pedicle graft	
- Omentum onlay graft	
- Rhomboid muscle	
- Latissimus dorsi muscle	
- Intercostal muscle	
T-tube drainage	
Drainage only	
Esophagectomy with	
- Immediate reconstruction	
- Delayed reconstruction	
Exclusion and diversion	

## Operative Management

Operative management is the main modality for treatment of esophageal perforation till today [61]. In the last 2 decades, the advances in anesthesia, postoperative care, total parenteral nutrition and powerful and selective antibiotics have caused substantial improvements in the outcome of esophageal perforation with surgical treatment [1-8]. There is no clear cut recommendation for indication of surgery but it includes: early postemetic perforation, hemodynamic instability, intra-abdominal perforation, extravasations of contrast into adjacent body cavities and presence of underlying malignancy, obstruction or stricture in the region of the perforation and surgically fit patient [1-8, 12, 14, 16]. All patients with esophageal perforation should undergo a planned intervention with an adequate period of resuscitation, and it should be done by the most experienced and a complete operating team in an elective list if possible [16, 17, 37]. The surgical procedure selected depends on surgeon’s experience, etiology of the perforation, time from injury to diagnosis and the site of perforation. Despite adequate surgical repair, continued esophageal leakage occurs in 30% of patients and 40% of these patients will require additional procedures there by increasing the morbidity and duration of hospitalization [35-37]. Different procedures described for esophageal perforation include primary repair with or without reinforcement [1-12], simple drainage of the thoracic cavity [21], exclusion diversion operation [22], and single stage esophageal resection with or without primary reconstruction [7, 8, 26-30]. Thoracoscopic repair using minimally invasive surgery is also described in the literature [70]. The fact that many procedures have been described in the literature is indicative that not a single surgical procedure could be considered a gold standard for the treatment of esophageal perforation [8]. Regarding the surgical procedure for delayed or missed rupture of esophagus is not very clear and still controversial. Preoperative preparation includes aggressive fluid resuscitation, control of sepsis by broad spectrum antibiotics and nasogastric intubation for gastric decompression. For early perforation the site of perforation and the status of the esophagus are important factors in deciding the type of surgery. Management of perforation in the healthy esophagus is different from perforation in a diseased or obstructed esophagus [8]. Resection of the esophagus is indicated in case of perforation in a diseased esophagus, whereas primary closure is indicated in perforation in a healthy esophagus. Perforations in the cervical esophagus are treated by primary closure and drainage of the neck. Upper thoracic esophageal perforations are approached by a right thoracotomy and left thoracotomy for the lower third. Lesions at the esophagogastric junction are approached by left thoracotomy or upper midline laparotomy. As a general principle all perforations require wide mediastinal drainage by opening the parietal pleura in its entire length of the esophagus [1-8]. Necrotic nonviable and grossly contaminated tissue in the mediastinum and the parietal pleura must be debrided. The esophagus and often the esophagogastric junction must be dissected completely to identify the site of perforation and mobilize the esophagus for a tension-free repair [1-8]. Whenever it is possible, the esophageal defect should be closed by primary suture repair, preferably in 2-layer closure of mucosa and muscularis. If it is not feasible, a single layer closure should be done. Some time it is not possible to do a direct closure because of friability of the tissue. In these cases, the esophageal tear closure should be done by using flaps over the defects [19, 58-65]. Various local tissues at the site of perforation have been used to buttress the primary repair [71, 78]. Pleural flaps, omental flaps, intercostal muscle flaps and pericardial flaps have been described [72-76]. The diaphragm flap has also been used for buttressing the suture lines after primary closure [78]. Regardless of the technique chosen, the use of buttress techniques has definitely improved the outcome of the surgical treatment [71-78]. Reinforcement with vascularized tissue decreases the fistula formation (13%) and mortality (6%), compared to repair without reinforcement (39%) and (25%) [72, 73]. All esophageal repairs should be drained by a large bore intercostal chest tube. A feeding jejunostomy should be always added for nutrition. Patients diagnosed with late perforations can usually be repaired primarily with reinforced muscle or pleura [19, 71, 78]. If primary repair is not possible because of the local tissue friability or there is severe mediastinitis, esophageal resection or exclusion and diversion should be considered [21, 22]. Exclusion and diversion comprises of cervical esophagostomy (diversion of the cervical esophagus and creating a salivary fistula), gastric decompression with a gastrostomy, esophagogastric junction stappling and jejunostomy [79]. Diversion procedures are relatively easy and quick procedures and should be performed early in patients with persistent sepsis despite initial surgical management, stenting as the initial step in patients unfit for a thoracotomy [79]. Drainage alone has been described for treatment of esophageal perforation, but it is still controversial [1]. Earlier reports suggest that drainage alone with other measure has got a worse outcome [59, 60]. Modified T-tube repair of delayed esophageal perforation results in a low mortality rate similar to that seen with acute perforations [80]. The use of esophagectomy for perforations was reported in the 1950s [81]. Patients with perforation of the esophagus in a diseased esophagus are best treated by resection [8, 82]. Esophageal resection with or without immediate reconstruction should be considered for perforations in patients with megaesophagus, carcinoma, caustic ingestion, stenosis or severe undilatable reflux strictures. If the underlying pathologic process is esophageal carcinoma, resection and immediate reconstruction are indicated if the lesion is otherwise resectable [7, 8, 26-30]. Postoperative care should be in critical care setting with haemodynamic monitoring, cardiac and respiratory support. Broad spectrum antibiotic should be continued for 7 - 10 days. Nasogastric decompression of the stomach until resolution of the postoperative ileus, after which enteral feeding should be started through a jejunostomy tube. Contrast study should be obtained on the 5th postoperative day to document the integrity of the repair. Long-term surveillance for stricture formation, reflux or carcinoma is also recommended (Table 3).

## Prognosis

In a recent review the overall mortality associated with esophageal perforation in 726 patients from series between 1990 and 2003 was 18% [83]. The mortality rate has not changed significantly compared to a mortality of 22% reported in a similar review of case series between 1980 and 1990 by Jones and Ginsberg [1]. Major prognostic factors determining mortality are the cause and location of the injury, the presence of underlying esophageal pathology, the delay in diagnosis and the method of treatment. Spontaneous esophageal perforation was associated with highest mortality of 36% (0% to 72%), followed by iatrogenic perforation with a mortality of 19% (7% to 33%), and traumatic perforation with a mortality of 7% (0% to 33%) [1-8, 16-37, 83, 84]. In relation to the location of perforation cervical esophageal perforations have better prognosis with a mortality of 6% (0% to 16%), whereas thoracic and abdominal perforations were associated with a mortality of 27% (0% to 44%), and 21% (0% to 43%), respectively [1-8, 16-37]. The interval from perforation to initiation of treatment is a crucial determinant of outcome after esophageal perforation. The overall reported mortality of esophageal perforation with treatment delayed by 24hours or more was 27% (0% to 46%) compared to 14% (0% to 28%) when treatment was initiated within 24 hours [1-8]. Amudhan et al [56] reported overall mortality 6.2% in their study group and none of the patients treated within 24 hours died and they concluded that early diagnosis decreases mortality and hospital stay in esophageal perforation. Eroglu et al [85] reported overall mortality of (11.4%); mortality among patients treated within 24 hours of sustaining the injury was substantially less than among those for whom diagnosis and treatment were delayed (3% and 36.4%, respectively). Cervical esophageal perforations resulted in lower mortality rates than thoracic or abdominal perforations. Patients treated by surgical management had less mortality then non operative management [85]. Patients underwent primary repair of esophageal perforation has better outcome then in patients with esophageal perforation repaired over a drain.

The mortality rate was 3% in primary repair group compared with 18% in patients with esophageal perforation repaired over a drain [61]. Recent literature favors primary repair as the best surgical approach with consistently low mortality ranging from 3% to 13% [86-88].

**Figure 1:**
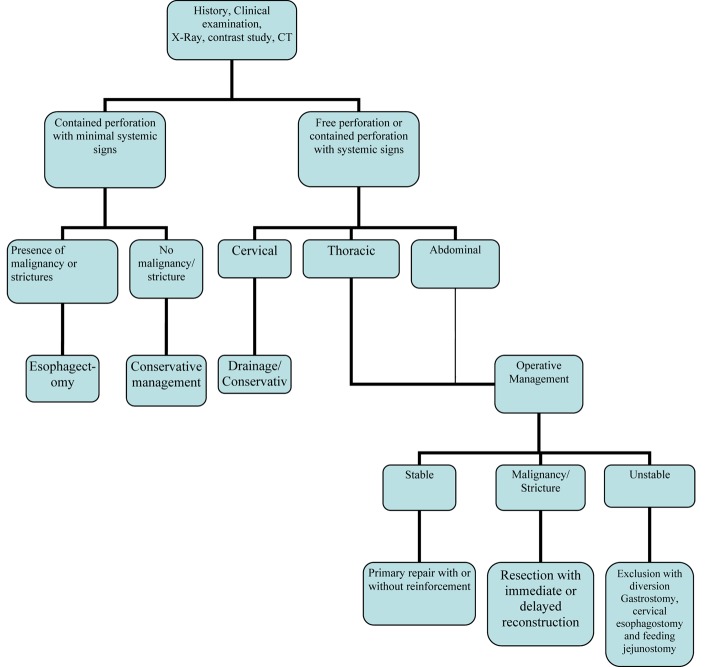
Management algorithm of esophageal perforation

In conclusion, esophageal perforation in adults is a highly morbid condition with high mortality. Mortality rates mainly depend on time of presentation and etiology of perforation. The overall mortality is 20%, while iatrogenic instrumental perforation has a lower mortality of 10%, and postemetic perforation has a higher reported mortality rate of 60-70%. The reported mortality from treated esophageal perforation is 10% to 25%, when therapy is initiated within 24 hours of perforation, but it could rise up to 40% to 60% when the treatment is delayed beyond 48 hours. The mortality rates are also higher in patients with thoracic and abdominal rupture and underlying esophageal disease like malignancy and benign stricture. For optimum treatment outcome an algorithm for management is suggested (Fig. 1).
